# Acid Balance, Dietary Acid Load, and Bone Effects—A Controversial Subject

**DOI:** 10.3390/nu10040517

**Published:** 2018-04-21

**Authors:** Lynda Frassetto, Tanushree Banerjee, Neil Powe, Anthony Sebastian

**Affiliations:** Deparment of Medicine, University of California, San Francisco, CA 94143, USA; tanushree.banerjee@ucsf.edu (T.B.); neil.powe@ucsf.edu (N.P.); anthony.sebastian@ucsf.edu (A.S.)

**Keywords:** osteoporosis, aging, kidney, alkali

## Abstract

Modern Western diets, with higher contents of animal compared to fruits and vegetable products, have a greater content of acid precursors vs. base precursors, which results in a net acid load to the body. To prevent inexorable accumulation of acid in the body and progressively increasing degrees of metabolic acidosis, the body has multiple systems to buffer and titrate acid, including bone which contains large quantities of alkaline salts of calcium. Both in vitro and in vivo studies in animals and humans suggest that bone base helps neutralize part of the dietary net acid load. This raises the question of whether decades of eating a high acid diet might contribute to the loss of bone mass in osteoporosis. If this idea is true, then additional alkali ingestion in the form of net base-producing foods or alkalinizing salts could potentially prevent this acid-related loss of bone. Presently, data exists that support both the proponents as well as the opponents of this hypothesis. Recent literature reviews have tended to support either one side or the other. Assuming that the data cited by both sides is correct, we suggest a way to reconcile the discordant findings. This overview will first discuss dietary acids and bases and the idea of changes in acid balance with increasing age, then review the evidence for and against the usefulness of alkali therapy as a treatment for osteoporosis, and finally suggest a way of reconciling these two opposing points of view.

## 1. Introduction

In western society, aging is accompanied by a loss of bone mass indicative of the bone disorder, osteoporosis. Osteoporosis is a condition in which bone resorption outpaces bone formation, leading to a combination of progressive loss of bone mass and destruction of bone architecture. The idea that osteoporosis might in part be due to dietary intake of acid precursors in excess of base (bicarbonate) precursors (i.e., a net acid-producing diet, or a diet net acid load) and that the consequent low-grade metabolic acidosis-induced bone mass reduction might be slowed or prevented by consuming a diet net base load was first proposed nearly 50 years ago by Wachman and Bernstein [1].

Modern Western diets, with higher contents of animal compared to fruits and vegetable products, have a greater content of acid precursors vs. base precursors, which results in a net acid load to the body [2,3]. To prevent inexorable accumulation of acid in the body (i.e., to prevent increasing positive acid balance) and progressively increasing degrees of metabolic acidosis, the body must be able to buffer, titrate, and/or excrete all of the excess acids or bases ingested or produced by their ingested precursors endogenously. Multiple systems in the body are part of the acid buffering and titration system, including the red blood cells, muscle cells, and bone. We define titration here as a process that does not regenerate itself, as compared to buffering, which does.

Bone is a large reservoir of base (25,000–30,000 milliequivalents (mEq) (1 milliequivalent equals 1 mmol of an ionic compound with a charge of +1 or −1, (e.g., sodium (Na), potassium (K), chloride (Cl), bicarbonate (HCO_3_))) in the form of alkaline salts of calcium (e.g., calcium hydroxyapatite, calcium carbonate). The concept that such bone base can be released to neutralize part of the dietary net acid load might be inferred from the finding from in vitro studies demonstrating that acids dissolve bone and from in vivo studies in animals and humans fed net acid-producing diets that bone dissolution occurs in conjunction with negative calcium balance (see references in Wachman and Bernstein [1]). This raises the question of whether decades of eating a high acid diet might contribute to the loss of bone mass in osteoporosis. If this idea is true, then additional alkali ingestion in the form of net base-producing foods or alkalinizing salts could potentially prevent this acid-related loss of bone.

Existing data currently supports both the proponents as well as the opponents of this hypothesis. Recent literature reviews by both groups tend towards those references that support their viewpoint. Assuming that the data cited by both sides is correct, we suggest a way to reconcile the discordant findings. This overview will first discuss dietary acids and bases and the idea of what can happen to acid balance with increasing age, then review the evidence for and against the usefulness of alkali therapy as a treatment for osteoporosis and finally suggest a way of reconciling these two opposing points of view.

## 2. Acid Balance

Balance in living systems means that the amount of substance going in equals the amount coming out, so that the levels inside the systems remain unchanging. Increased or decreased intake or production will raise or lower systemic levels, respectively. In the same vein, decreased or increased excretion will raise or lower systemic levels, respectively.

In humans, blood pH levels reflect the net production, buffering, and excretion of all of the acids and bases in the system. And there is a range—that is a spectrum—of pH levels considered to be normal. Young humans with completely normal kidney function maintain their blood pH and bicarbonate levels at the higher end of the spectrum. Thus, even with high diet acid intakes, their kidneys are able to excrete the excess acid in the urine.

With increasing age, the body’s ability to maintain blood pH levels declines, so that by the 9th decade, blood pH and bicarbonate levels have now fallen to the lower end of the spectrum [4]. The most likely reason for this is due to declining kidney function with age, since renal net acid excretion contributes the largest amount to the elimination of non-carbonic (i.e., not H_2_CO_3_) acid, through generation of new bicarbonate delivered to the systemic circulation. To put this in terms of acid/base balance, net acid excretory ability has decreased and if acid/base intake remains the same, the balance becomes positive and the total acid content inside the system has increased. Similarly, if excretory ability remains unchanged, increased intake or production of acid will also raise systemic acid levels.

## 3. Dietary Acids and Bases

Food and endogenous metabolic processes are the sources of acid or base intake or production. Studies on the effects of diet on urinary pH and acid excretion to alter acid-base balance started at the end of the 19th century [5,6,7]. Subjects in these early studies would be fed specific diets and the urine analyzed for nitrogen compounds such as urea, non-urea nitrogen, and ammonia, as well as sulfates, phosphates, and chlorides.

In the late 1950s, the pioneering group of Relman, Lemann, and Lennon undertook an impressive series of landmark studies [8,9,10]. Evaluating both liquid and solid diets, they investigated the correlation between endogenous acid production and renal acid excretion. They showed that the net acid production was the sum of (1) the net liberation of protons from organic phosphate compounds, (2) the oxidation of organic sulfur to sulfates and (3) the endogenous formation of unmetabolized organic acids [11]. Dietary base was produced from the ingestion of organic anions such as citrate or malate, which are metabolized to bicarbonate. Much of the bicarbonate is excreted by the lungs in the form of carbon dioxide. In 1966 [12], the group was able to demonstrate that knowledge of both the composition of the dietary precursors and the metabolic end products excreted in the urine and feces (Net endogenous acid production = urinary organic acids + sulfates-bicarbonate; Net renal acid excretion = urinary ammonium plus titratable acids minus bicarbonate) was required to calculate the quantity of “fixed” or non-carbonic acids produced from a given diet.

Biochemical analyses of food demonstrate that almost all foods contain acid precursors, while fruits and vegetables also contain base precursors. Using this information, dietary formulas for estimating the acid or base effects of different foods have been developed [13,14,15]. Use of estimates of dietary intake avoids having to measure renal net acid excretion. However, these formulas require quantitative analyses of both dietary cations (sodium, potassium, calcium, magnesium) and anions (chloride, sulfate, phosphate). The formulas may also contain an estimate of organic anion production and/or a factor for intestinal ion absorption. Typical western diets produce approximately 1 milliequivalent of dietary acid per kilogram body weight, or approximately 50 mmol of acid/day [16].

## 4. Proponents of the Alkali Replacement for Prevention of Osteoporosis Theory

Proponents of the alkali treatment for osteoporosis theory argue that in vitro studies demonstrate that large increases in system acid levels first activate osteoclasts, which increases calcium flux from bone, contributing to increased bone dissolution [17,18,19,20,21]. Therefore, smaller changes that promote higher blood and tissue steady-state acid levels in in vivo systems, produced over a lifetime of chronic ingestion of typical net acid-producing diets, exacerbated by the age-related decline in the kidney’s ability to completely regulate acid-base balance, might become a clinically significant contributor to osteoporosis. In healthy humans, the normal range for blood pH is between 7.35 and 7.45 [22]. The in vitro studies by Arnett and Dempster demonstrated that osteoclasts, the bone cells that dissolve bone, are activated at low pH (around 7.0) and become progressively less active as the pH approaches 7.4. Conversely, osteoblasts, the bone cells that deposit new bone, work best at a pH of 7.4 and in a more acid environment will not mineralize bone collagen matrix. Interstitial tissue pH has been measured between 7.0 and 7.2 [23]. Bushinsky and his colleagues have elegantly demonstrated that decreasing system pH increases the release of calcium from both live (i.e., with osteoclasts) and dead bone, indicating that at low system pH, physiochemical processes also cause bone dissolution. They have linked systemic acid levels to activation of the G-protein coupled hydrogen ion receptor OGR-1, found in both osteoblasts and osteoclasts [24].

Proponents of the alkali therapy for osteoporosis treatment then cite human studies that support this hypothesis [25,26,27,28,29]. These studies include short term studies, looking at markers of bone formation and bone breakdown and longer term studies, evaluating bone mineral density by imaging studies.

In the study conducted by Sebastian et al. [28], 18 postmenopausal women given oral administration of potassium bicarbonate (KHCO_3_) for 18 days at a dose sufficient to neutralize endogenous acid (from 70.9 ± 10.1 to 12.8 ± 21.8 mmol per day) demonstrated improved calcium and phosphorus balance—that is, less was excreted in the urine and stool in comparison with the amount ingested (mean ± SD (standard deviation); change in calcium balance, +56 ± 76 mg (1.4 ± 1.9 mmol) per day per 60 kg, *p* = 0.009; change in phosphorus balance, +47 ± 64 mg (1.5 ± 2.1 mmol) per day per 60 kg, *p* = 0.007). This was associated with reduction of the bone resorption marker urinary hydroxyproline (from 28.9 ± 12.3 to 26.7 ± 10.8 mg per day (220 ± 94 to 204 ± 82 μmol per day, *p* = 0.05). And increase in the bone formation marker serum osteocalcin concentrations (from 5.5 ± 2.8 to 6.1 ± 2.8 ng per milliliter, *p* < 0.001). Although this trial was carried out in a relatively small selected group of white women who were physically active and had undergone menopause at least five years earlier, the trial was carried out under strictly controlled conditions in a clinical research center. The findings of this trial were consistent with the current knowledge of the acid-base responses of osteoclasts and osteoblasts studied in vitro.

A 4 week randomized, controlled study in 30 young women with a normal calcium intake compared the effect on bone markers of an alkaline mineral water, rich in bicarbonate, with that of an acid one, rich in calcium only. [26] In these otherwise healthy young women, the acid calcium-rich water had no effect on bone resorption while the alkaline water rich in bicarbonate led to a significant decrease in serum parathyroid hormone and the bone resorption marker C-telopeptide. This study had limitations, including the number of subjects being small, which might limit statistical power to detect more effects. However, the findings were consistent with other studies that have also shown a decrease in bone resorption through nutritional alkaline load, either by potassium bicarbonate [28], an alkali diet with bicarbonate-rich mineral water [30] or K-citrate [29].

So, in short term studies, alkali therapy lowered bone resorption markers. Short term studies, however, cannot be used to evaluate changes to bone density.

Jehle et al. [29] carried out a 2-year randomized, double-blind, placebo-controlled trial comparing the effects of 60 mmol of K-citrate vs. placebo on age-associated decline in bone mass and bone architecture (bone quality) in healthy elderly participants. The study participants were non-vegetarian men and women in the age range of 65–80 years. The main study endpoint was the change over 24 months in areal bone mineral density (aBMD) at the lumbar spine by dual-energy X-ray absorptiometry. The investigators also evaluated changes in volumetric density and microarchitectural parameters by quantitative computerized tomography.

The findings demonstrated a significant increase from baseline values in aBMD (at lumbar spine, +1.7 ± 1.5%, *p* < 0.001) and volumetric BMD (trabecular volume, thickness and number, *p* < 0.05 for all three) in association with long-term suppression of diet-induced acid loads. Further, according to the Fracture Risk Assessment (FRAX) prediction model, K-citrate significantly decreased fracture risk prediction after 24 months when compared with baseline and compared with the placebo group. This study, although being a placebo-controlled trial, was a single center study and had an unrepresentative population limited to Caucasian participants living near the study site. Despite the noted limitation, the effects of K-citrate on BMD were similar in both genders and were consistent with the similar longitudinal rates of decline in fracture risk reported in elderly Caucasian men and women as noted in the studies carried out by Melton et al. [31] and Kaptoge et al. [32].

## 5. Opponents of the Alkali Replacement for Prevention of Osteoporosis Theory

Opponents of this theory make the argument that if bone were the main source of base used to titrate dietary acids, then in just a few years, all the bone in the body would dissolve. They argue that since this does not occur, bone cannot be the major site of base (alkali) for diet net acid neutralization. They further argue that the results of studies demonstrating positive acid balance could be due to errors in measurement techniques for acid excretion [33,34,35].

Elegant quantitative analyses by Oh and Uribarri in subjects with chronic metabolic acidosis (where the blood pH is often below the normal lower limit of 7.35) argue that the 12–19 mmol of acid per day that these subjects retain would need to be buffered by an equal amount of base. Bone made up of calcium hydroxyapatite contains ~25,000 mmol of alkali and at the rate of 19 mmol/day, the alkali supply would be exhausted in less than 4 years. Instead, they argue that production of both organic acids and sulfuric acid is decreased in advanced renal failure and the rest of the acids are eliminated in some unknown and therefore unmeasured form.

The opponents of the alkali therapy for osteoporosis treatment cite other supporting human studies [36,37,38,39,40]. These studies include meta-analyses of cohort data and epidemiological studies, generally in healthy subjects.

Macdonald et al. [36] conducted a 2-year randomized placebo-controlled trial in 276 postmenopausal women aged 55–65 years. Subjects were randomized to 4 groups: high-dose K-citrate (55.5 mEq/day), low-dose K-citrate (18.5 mEq/day), placebo, or 300 g additional fruit and vegetables per day (equivalent of 18.5 mEq alkali). This study was able to conceal the allocation to the study groups by concealing the process and therefore is of quality. The results showed no persistent changes in bone turnover or bone mineral density over two years with a daily dose of 18.5 or 55.6 mEq K-citrate or with self-selected fruits and vegetables per day, which is more likely to be an accurate reflection of the truth since the randomized design is less likely to be biased. A transient reduction of the bone breakdown marker free deoxypyridinoline cross-links relative to creatinine was noted only in the high-dose K-citrate group at 4–6 weeks, but that value returned to baseline at 3 months. This was one of the longer studies investigating the influence of dietary alkali on bone health; it used bone turnover assessments at regular intervals and studied bone density using standard Dual Energy X-ray Absorptiometry (DEXA) measurements.

Dietary sodium, chloride, and potassium alkali can alter blood pressure as well as alter dietary acid balance [41]. A retrospective two-center analysis of 266 otherwise healthy postmenopausal women given alkali therapy for at least 2 years by Frassetto et al. [37] demonstrated no relationship of blood pressure responses to Na, Cl, or K intake and bone mineral density response to diet alkali therapy. Mean arterial pressure (MAP) was calculated from blood pressure measures, divided into tertiles and its influence on the effect of dietary NaCl and K alkali supplementation on bone mineral density was tested. No effect of dietary alkali treatment on BMD change or bone resorption was shown for either center. Adjusting for the possible calcium- or potassium-lowering effects on blood pressure or MAP did not alter the results.

Thus, in these two relatively long-term randomized, controlled trials in mostly older Caucasian women who would be expected to be at risk for lower bone mass, neither study demonstrated significant changes in bone density.

Fenton et al. [38] with superior methodology carried out a meta-analysis to assess the effects of changes in net acid excretion from changes in diets or “alkaline” supplements on both urine calcium and calcium balance in studies of calcium metabolism. These studies, investigated alterations to calcium balance as a result of alterations to either the quantity and/or the type of protein. Analyses demonstrated a significant linear relationship between an increase in urinary net acid excretion (NAE) and urinary calcium (*p* < 0.0001), but no changes in calcium balance (*p* = 0.38; power = 94%). No relationship was noted between a change of NAE and a change in the marker of bone resorption, N-telopeptides (*p* = 0.95). This meta-analysis had sufficient power because variability between subjects was eliminated by the use of cross-over designs in the included studies and systematically assessed the calcium balance literature in response to changes of NAE. Calcium balance is a surrogate measure of bone disease progression but does not directly measure either bone health or changes to bone health.

The study findings were corroborated by the population-based Canadian Multicentre Osteoporosis Study (CaMOS) which examined associations between urine acid excretion and osteoporosis [39]. Adjusting for confounders, the CaMOS study demonstrated no correlations of either urine pH or acid excretion with the incidence of fractures (6804 person years) or changes over 5 years in bone mineral density. This study examined change in BMD and occurrence of fragility fractures as the outcome measures, which are superior clinical outcomes measures of osteoporosis relative to the measurement of urine calcium changes. The authors used urine to measure the diet acid load to avoid the random and systemic errors inherent in food intake measurement. The exposures for this study were measured using fasting morning urine samples. While it is possible urine pH in 24 h sample might have a relationship with bone outcomes, fasting morning urine pH does not correlate with 24 h net acid excretion [42].

Thus, even in these larger scale studies, the authors found no evidence of an effect of diet acid load on not just bone density, but fracture incidence.

## 6. Controls on Physiologic Acid-Base Regulation

To review, arguments from studies in subjects with advanced renal failure and the results of current epidemiological literature examining the potential association between dietary acid load and bone density and fracture incidence are conflicting to results found from in vitro studies and at least one longer term randomized alkali treatment study in humans. Is it possible to reconcile these two disparate points of view? We believe there are at least four points of discussion: the magnitude of the effects of acid on bone in healthy living systems, whether high diet acid loads do indeed increase the net balance of acid in the system at steady state, the presence of other acid regulatory systems besides bone and changes in the buffering capacity of the body’s tissues (e.g., muscle, kidney) associated with the inability of the aging kidneys to maintain their previous acid excretory ability.

The negative effect on bone of habitual consumption of typical net acid-producing diets may be relatively small. For example, age, gender, weight, and immobility are likely to have a quantitatively greater effect on fracture risk than coffee drinking, smoking, or trace mineral levels [25,43]. In the Study of Osteoporotic Fractures analyses, after adjusting for age, weight, estrogen use, tobacco use, exercise, total calcium intake, and total protein intake, elderly women on higher acid diets had a higher rate of bone loss and greater risk of hip fracture than those on low acid diets [25]. Diet acid load may be another extant, but quantitatively small factor.

In healthy subjects, higher dietary acid loads do lead to higher steady state blood acid levels, within the range considered to be normal. Both Kurtz et al. [3] and Frassetto et al. [44] using steady state diet data from healthy humans admitted to a metabolic balance ward, eating diets whose net acid loads were within the range typically observed in American and European diets, showed blood hydrogen ion concentration was significantly higher and the plasma bicarbonate concentration significantly lower. The body’s homeostatic mechanisms were unable to maintain previous hydrogen ion or bicarbonate levels with diet acid loads greater than ~1 mmol/kg/day.

Higher steady state acid levels have been shown to increase with increasing age [4]. In a meta-analysis of 971 otherwise healthy subjects at steady state, blood pH decreased about 7% associated with a decrease in steady stage plasma bicarbonate of about 12% as age increased from 20 to 80 years.

Otherwise healthy people also tend to have a decline in renal function as they age [45]. This is associated with a reciprocal increase in steady state blood acid levels [44]. With increasing decline in renal function, blood acid levels continue to increase [46,47]. Wesson and colleagues have also demonstrated that the tissue buffering capacity of the kidneys of rats with 5/6 nephrectomy was decreased in comparison to the kidneys of sham operated rats [47].

In patients with chronic kidney disease stage 2 (CKD 2), who have a degree of renal insufficiency similar to that seen in older subjects with age-related decline in kidney function, Wesson et al. demonstrated reduced acid excretory ability and consequent tissue hydrogen ion retention compared to subjects with CKD stage 1 [48,49,50]. This group has also recently demonstrated decreased urinary citrate excretion in these subjects, consistent with the idea of a decreased acid excretory capacity [51]. Of perhaps equal importance is that data suggests that high tissue acid levels promote the progression of renal damage [52]. Both in subjects with advanced CKD and in subjects with CKD stage 2, treatment with bicarbonate slowed progression of decline in Glomerular Filtration Rate (GFR) and initiation of dialysis [48,49].

Another potential system for titrating acids was suggested by Hood and Tannen in 1998. They hypothesized that systemic pH was protected by increasing or decreasing the production of organic acids, in the direction that mitigated the effect on systemic pH [53]. Studying overweight humans who were fasting or on ketogenic diets, they demonstrated that the subjects given an acid (ammonium chloride) had both lower levels of blood ketoacid production as well as urinary ketoacid excretion. These effects would tend to limit the net amount of additional acid in the system. Those given sodium bicarbonate had both increased blood ketoacid production as well as urinary ketoacid excretion, thereby increasing blood acid levels. Thus, quantitative changes in organic acid production may be one of the main methods by which the body adjusts and maintains systemic blood acid levels.

## 7. Synthesis

We suggest the way to put all of this information together is to agree with Oh and Uribarri that bone alone cannot buffer the positive acid balance that one can calculate from measures of acid production and acid excretion. In addition, endogenous acid production goes up and down in an attempt to maintain systemic blood pH. Younger subjects with better renal function are able to maintain their blood pH in the higher range of normal, while older subjects with worse renal function are only able to maintain their blood pH in the lower ranges of normal. As renal function declines, renal acid production goes down, but not as much as renal acid excretion and the tradeoff is that the blood acid levels and therefore acid balance are higher. These higher acid levels appear themselves to lead to more rapid damage to the kidneys [48,49].

Diets high in acid precursors add to the body’s acid burden. For the majority of people eating typical western diets with acid loads of ≤1 mmol/kg, whose renal function and acid excretory ability is normal, dietary acid loads would not be a readily detectable factor in altering bone mineral density leading to the development of osteoporosis. Other factors such as age, gender, race, and immobility are quantitatively more major factors in determining bone mass and bone breakdown.

However, body retention of only 1 or 2 mEq of acid each day, barely detectable by current measurement techniques, buffered by muscle and kidney and titrated by skeletal base over decades, could potentially result in major depletion of bone mineral. Thus, we suggest that those older subjects with diminished renal function, decreased renal acid excretory ability, and lower buffering capacity due to lower muscle and/or bone mass, whose diets contain high net acid loads could potentially benefit the most from alkali therapies.
